# Validating the concept of top scavenger: the Andean Condor as a model species

**DOI:** 10.1098/rsos.240409

**Published:** 2024-07-31

**Authors:** Diego Méndez, Félix Hernán Vargas, José Hernán Sarasola, Pedro P. Olea

**Affiliations:** ^1^ Departamento de Ecología, Universidad Autónoma de Madrid, Calle Darwin 2, Madrid 28049, Spain; ^2^ Aves Rapaces en Bolivia – Programa de Investigación. Calle El Villar 369, Sucre, Bolivia; ^3^ The Peregrine Fund – Programa Neotropical, Calle Pingüino 52, Isla Santa Cruz, Galápagos, Ecuador; ^4^ Centro para el Estudio y Conservación de Aves Rapaces en Argentina (CECARA), Universidad Nacional de La Pampa, Avda Uruguay 151, Santa Rosa, La Pampa 6300, Argentina; ^5^ Instituto de las Ciencias Ambientales y de la Tierra de La Pampa (INCITAP-CONICET),Avda. Uruguay 151, Santa Rosa, La Pampa 6300, Argentina; ^6^ Centro de Investigación en Biodiversidad y Cambio Global (CIBC-UAM), Universidad Autónoma de Madrid,Calle Darwin 2, Madrid 28049, Spain

**Keywords:** avian scavenger, carcass consumption, carrion removal, domestic dog, scavenger guild, *Vultur gryphus*

## Abstract

Vultures provide the key ecosystem service of quickly removing carrion, so they have recently been assumed to be top scavengers. To challenge the concept of top scavenger (i.e. the most influential in the scavenging community and process), between 2012 and 2019, we recorded the consumption of 45 equine carcasses available for two different avian scavenger guilds in the Tropical Andes; each guild included the Andean Condor, the alleged top scavenger. The carcasses eaten by Andean Condors were consumed, on average, 1.75 times faster than those they did not eat. Furthermore, the greater abundance of feeding condors shortened carcass consumption time more than a greater abundance of any other species by 1.65 to 5.96 times, on average. These findings support the hypothesis that the Andean Condor significantly drives scavenging dynamics and is, therefore, an unrestricted top scavenger. Additionally, we established a gradient of tolerance of avian scavengers to domestic dog disturbance at carcasses, from highest to lowest: vultures > caracaras > condors. Our study framework holds great potential for advancing in food webs’ comprehension through quantifying the relative functional role of scavenging communities’ members and for guiding efforts to weigh up the ecological contributions of top scavengers and foster their conservation.

## 1. Introduction

In ecology, the definition of apex or top species is generally associated with the species’ position in the highest level of food chains or their predominant ecological roles; that is, they are species not preyed upon by other species or species that functionally dominate their guilds. Thus, top species typically are large species whose presence in ecosystems can have cascading effects throughout ecological levels, for example, by regulating the populations of other species, or impacting vegetation composition and structure [1,2]. Notably, while large predators have been widely recognized as top-tier species [1], it has been less common to extend this concept to non-predatory species high in food chains, such as scavengers [3]. This may be because in both theoretical and empirical research on food webs, scavenging and scavengers have traditionally been overlooked [4], even despite their significant contribution to ecosystem stability and functioning by favouring major energy transfer [5]. Vultures, the quintessential scavengers, exemplify this situation [6].

Vultures, the only known obligate scavengers, are a highly specialized functional group of birds that includes 22 species: the seven New World vultures (Cathartidae) and 15 of the 16 species of Old World vultures (Accipitridae), the exception being the Palm-nut Vulture *Gypohierax angolensis*, which is mainly frugivorous [7]. Thanks to their ability to fly effortlessly, keen senses, social foraging behaviour, large bodies, sharp beaks and nude heads, vultures are outstandingly efficient in detecting and consuming carrion [7,8]. They also stand out because they face several threats, often exacerbated by their idiosyncratic foraging strategy, whereby many individuals can gather to feed in a single location, simultaneously exposing themselves to a given threat [8]. Among their most serious threats are poisoning and, of particular interest with respect to the functioning of scavenger assemblages, competition for food with domestic dogs (*Canis lupus familiaris*) [6,9]. Domestic dogs disrupt animal communities throughout the planet [10] and can become unwanted dominant scavengers, directly disturbing vultures and their role [11–13].

Along with being highly threatened, vultures are so well adapted for a scavenging lifestyle that they are capable of modulating carrion consumption, structuring scavenger assemblages and effectively contributing to keeping the environment free of potentially polluting or pathogenic matter; hence, their ecological role is considered unique and irreplaceable [6–8]. Indeed, the fact that vultures have such a dominant influence on the use of carrion and the ecological interactions around it, disproportionate compared to that of other scavenger species [14–17], has led to them being referred to as top or apex, scavengers [e.g. 14,18–23].

Interestingly, the term top/apex scavenger has not been applied uniformly to all vultures. Thus, although this term began to be used anecdotally to refer to the Andean Condor (*Vultur gryphus*) [24], a New World vulture, it is noteworthy that it has not been used to refer to the rest of the vulture species in the Americas. Similarly, in the case of Old World vultures, the term has been used mainly to refer to a single species, the Griffon Vulture (*Gyps fulvus*) [14,19–23], with the difference that its consideration as a top scavenger appears to have been more generally extended to the rest of vulture species [18]. Considering this, what seems to be a conceptually assumed rather than formally evaluated designation of vultures as top scavengers may be owing to several reasons. In the case of the Andean Condor, its size and strength, notably greater than those of co-occurring scavengers, gives it an advantage in accessing and defending carrion and, therefore, a dominant status within the scavenger guild [25,26]. This may have led to its scavenging role being taken for granted, with it being defined as a top scavenger likely on the basis that it is the largest and can potentially consume the most food [3]. On the other hand, the Griffon Vulture has been, broadly speaking, more studied than any other vulture species [27]; thus, it might be thought that its role as a top scavenger is based on extensive evidence; however, this is not the case [18]. In view of this, and considering that an informed delineation of functional groups is essential to investigate, manage, and conserve them [28], it can be concluded that explicitly examining vulture attributes as top scavengers is a useful and timely approach to enrich our theoretical and practical knowledge about them, which is particularly necessary for New World vultures [29,30].

In this context, the Andean Condor represents an ideal case study to deepen the understanding of the role of top scavengers within their guilds, while enlightening scavenging dynamics where this species occurs. For instance, avian scavenger guilds in the Andes are characterized by including a single large species (i.e. condors) and a certain number of smaller size-varying species of both obligate and facultative scavengers (e.g. vultures and raptors). They find carcasses using sight and, some of them (*Cathartes* vultures), also smell, and avoid competition primarily by establishing dominance hierarchies among individuals at carcasses [7,8,25,31]. On this basis, it would be particularly revealing to assess aspects such as scavenger guild composition, carcass detection and consumption times for each consumer species, species’ abundances at a carcass and its relationship with the time it takes for a carcass to be consumed [17,32,33].

Seeking to address this topic, we used observer-placed large carcasses (domestic equines) to study the relative scavenging role of the Andean Condor across the eastern Andes of Bolivia, central South America, one of the few locations in the Andes where up to four species of New World vultures coexist [31]. Likewise, we took the opportunity to assess the threat that domestic dogs pose to the Andean Condor [34], as well as the influence of these mammals on carcass consumption and their interactions with avian scavengers.

In order to define the Andean Condor as a top scavenger (table 1), we aimed to answer how fast this species was in detecting and starting to consume single carcasses, how bold it was to approach and feed on them, and how abundant it was when feeding on them in relation to other avian scavenger species within two guilds, one of five species and another of three. Accordingly, we hypothesized that the Andean Condor should excel in carcass consumption regardless of the guild of which it is part, and that its abundance should be more influential on the time it takes for a carcass to be consumed than the abundance of other avian scavengers. Finally, we offer insights into the impact of domestic dogs as stressors on the functionality of the Andean Condor as a top scavenger of scavenger assemblages in the Andes.

**Table 1.** Fittingly analogous to the most popular of sports expressions—Citius, altius, fortius—Communiter (Faster, higher, stronger—Together), which transcends its primary interpretation by also referring to the way in which a specific task or activity should be carried out, as a community [35,36]—a parsimonious checklist of the attributes of a top scavenger is postulated.

attribute	rationale for top scavenger species	the Andean Condor, a top scavenger
faster	a top scavenger significantly reduces the time in which carrion is consumed	Andean Condors take advantage of social foraging to quickly find a carcass, and each of them is capable of eating up to at least 3 kg of food in one sitting [7,8]. Furthermore, since they usually form large feeding groups [37], they have the potential ability to quickly consume any carcass.
higher	it fulfils its scavenging role on a large spatial scale, significantly larger than that of other scavengers, both at the individual and population levels, being able to detect and access a high proportion of available carcasses	With home areas of up to c. 50,000 km (authors’ own data), Andean condors are probably the non-migrant vulture species that show the greatest movement capacity of all. This, accompanied by the well-known visual capacity of vultures [7,8], makes it more likely that condors will detect and access a greater number of carcasses.
stronger	it is able to access (i.e. rip open and tear apart) and consume any type of carrion available, with a body size that allows it to exert physical dominance over the rest of the scavengers	Weighing between 8 and 15 kg, it greatly exceeds any other avian scavenger in size. Within its distribution range, it is followed in size by the King Vulture (3–4 kg weight), followed by the rest of the obligate and facultative avian scavengers (around 2.5 kg weight) [31]. Therefore, it is unlikely that any avian scavenger has a chance of displacing a condor in a one-to-one situation. Culpeo foxes (Lycalopex culpaeus) (7–13 kg weight) are the largest scavenging mammals throughout the Andes and, although it is uncommon to see them feeding alongside condors when they do, it is also uncommon for them to interfere with each other, noting that when condors are found in large numbers, culpeos generally stay away from carrion, just as other scavengers do. Furthermore, a male Andean Condor may be able to open a hole in the toughest part of the hide of a cow carcass with less than 15 pecks (own observations).
together	it is an axis for coexistence among scavengers by being able to structure competition and facilitation, irrespective of the scavenger species it interacts with	Regardless of how many condors feed, their participation facilitates (e.g. by opening a large carcass and allowing access to other smaller scavengers) or limits (e.g. by almost monopolizing a carcass thanks to its number, size and strength) the participation of other scavengers. In this way, it can regulate carrion consumption in the communities and ecosystems of which it is part.

## 2. Methods

### 2.1. Study area and species recorded

**Figure 1.**
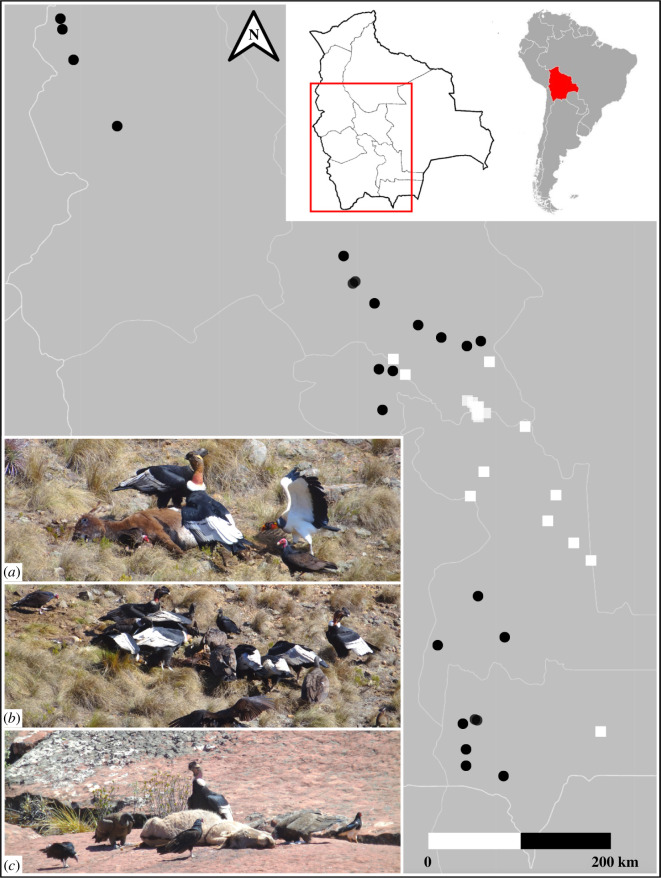
The study area (red outline on the inset map) extended through central South America along Bolivia (the country in red) in a mainly north–south direction. Black dots (*n* = 21; carcasses available for the five-species avian scavenger guild) and white squares (*n* = 24; carcasses available for the three-species one) show the distribution of placed carcasses. Boxes (*a*) and (*b*) (photos from the same carcass site) show the species exclusive to the five-species guild (King and Black vultures; and Southern Caracara), box (*c*) shows the species exclusive to the three-species guild (Mountain Caracara). The Andean Condor and the Turkey Vulture appear in the three boxes.

This study, conducted between 2012 and 2019, was developed from field work aimed at obtaining population estimates of the Andean Condor (during 2012 and 2014 [38,39]); and at capturing Andean condors and King vultures (*Sarcoramphus papa*) for subsequent telemetry monitoring (during 2018 and 2019) which included the placement of 45 equine carcasses, ethically sourced from local farmers, along the eastern Andes of Bolivia between 14° 41' S and 22° 2' S, between 63° 39' W and 69° 7' W and at elevations between 1200 and 5000 m asl. (figure 1; electronic supplementary material, S1). The avian scavenger species that occur there are the Andean Condor, the King, Black (*Coragyps atratus*) and Turkey (*Cathartes aura*) vultures and the Southern (*Caracara plancus*) and Mountain (*Phalcoboenus megalopterus*) caracaras. In addition, records of two facultative canid scavengers—the Culpeo fox and the domestic dog—were also obtained.

### 2.2. Avian scavenger guilds, and carcass categorization and covering

Based on the distribution ranges of the avian scavengers in our study area [31], 21 carcasses were potentially available to condors, King, Black, and Turkey vultures, and Southern caracaras in the mid-altitude Andes (mainly below 3000 m asl); while 24 were potentially available to condors, Turkey vultures and Mountain caracaras in the higher Andes (mainly above 3000 m asl). Accordingly, we categorized the carcasses into two groups: the group of carcasses available to the five-species guild (*n* = 21) and the group of carcasses available to the three-species guild (*n* = 24) (figure 1; electronic supplementary material, S1).

All the carcasses were potentially available to both culpeos and domestic dogs, the two species being mostly nocturnal or crepuscular foragers. Since we did not collect any data or watch the carcasses during the night, and that dogs’ presence would have distorted or handicapped the purpose of our field work, we covered the carcasses overnight in those places we considered most likely to be visited by them (i.e. places less than 5 km away from the nearest village or area of human activity such as livestock herding or crop care), in order to reduce that likelihood. Using a heavy canvas, we covered 13 carcasses (61.90%) of the five-species guild and 14 carcasses (58.33%) of the three-species guild. This prevented us from recording nocturnal scavenging and conditioned our ability to make unbiased observations of culpeos and domestic dogs. However, we did analyse differences in the consumption of covered and uncovered carcasses, and of carcasses that dogs visited during the day and those that did not, given the relevance of these comparisons for the objectives of this study.

### 2.3. Field procedures

The 45 equine carcasses (23 donkeys and 22 horses, weighing c. 200 kg each) were placed, one by one, in 2012 (10 carcasses), 2014 (27 carcasses), 2018 (4 carcasses) and 2019 (4 carcasses) throughout the study area in open spots, equally detectable and accessible to avian scavengers (i.e. flat non-forested places where no habitat element could cover the carcass, near or visible from natural perches for avian scavengers) (electronic supplementary material, figure S1). Carcasses were placed before dawn and each was monitored by at least two observers from hides distant 35–100 m from the carcass. Observations were made daily from 7.00 to 19.00 h (local time), from the day the carcass was placed until it was totally consumed (i.e. only skin and bones were left), making a total of 185 days (22.20 h) of observation.

### 2.4. Data collection

At each carcass site, we classified each avian scavenger species into one of three carcass use categories: (i) visited and fed, (ii) visited and did not feed and (iii) did not visit. Two main types of data were collected, namely counts of avian scavenger species and individuals, and the following time measurements in hours: carcass detection time (by avian scavengers), carcass consumption start time (by avian scavengers) and carcass consumption time.

#### 2.4.1. Counts

Every 10 min, we counted the number of avian scavengers that were at the site (flying or on the ground) and recorded the local abundance of each species as the maximum number of individuals observed feeding at the same time (i.e the number of individuals of each species that we were certain was involved in consuming the carcass over its total observation period). Therefore, when a species did not visit a carcass or visited it but did not feed, its abundance was recorded as zero.

#### 2.4.2. Time measurements

Carcass detection time of each avian scavenger species was recorded as the time elapsed between carcass placement and the moment when an individual of the corresponding species was observed to unequivocally approach or inspect the carcass, counting daylight hours only. The carcass consumption start time of each avian scavenger species was recorded as the time elapsed between carcass detection and the moment an individual of the corresponding species started to feed, counting daylight hours only. Carcass consumption time was recorded as the time elapsed from carcass placement until it was totally consumed.

### 2.5. Data analysis

Data were analysed to compare the scavenging roles of avian scavengers within guilds, between guilds (in the case of species that were present in both guilds; that is, Andean condors and Turkey vultures), or considering the two guilds as a whole, as required to answer our research questions and test the proposed hypotheses [40,41]. Within guilds, we used Fisher’s exact tests to compare the proportions in which the species used the carcasses (i.e. those that visited and fed on, only visited or did not visit). We also used Fisher’s exact tests to compare the proportions in which Andean condors used carcasses in the five-species guild versus in the three-species guild and did the same for Turkey vultures, as the two species were present in both guilds. Within guilds, we used Kruskal–Wallis tests followed by Dunn–Bonferroni corrections to compare species abundances. We used Mann–Whitney tests to compare the abundances of Andean condors and Turkey vultures between guilds.

To compare carcass detection time and carcass consumption start time among species within each guild, we fitted two generalized linear mixed models (GLMM) with Gamma distribution and log link function per guild, each model involving one of the two-time measurements as the response variable and species identity (five levels in the five-species guild; three levels in the three-species guild) as the predictor (fixed effect). Carcass (ordinal variable) was included as a random term in all the GLMMs in order to control for the non-independence of data collected through multiple records in the same carcass. Likewise, and because they were present in both guilds, we compared carcass detection time and carcass consumption start time of condors and Turkey vultures across carcasses. For each species, we fitted two generalized linear models (GLM) with Gamma distribution and log link function; each of these two models involved one of those time measurements as the response variable, and both models involved the guild (two levels: ‘five-species guild’ and ‘three-species guild’) as the predictor.

To examine the relative role of avian scavengers in carcass consumption, with a focus on the Andean Condor and considering the influence of nocturnal scavenging and domestic dogs, we followed three approaches. First, we assessed the relationship between carcass consumption time and the abundance of avian scavengers in each guild, by fitting a GLM with Gamma distribution and identity link function to the data from each of them. Both models involved carcass consumption time as the response variable and the abundance of each avian scavenger species (five and three species in the five-species guild and the three-species guild, respectively) as the predictors. Second, we fitted a GLM with Gamma distribution and inverse link function to the data from all carcasses, which also involved carcass consumption time as the response variable and Andean Condor’s carcass use (two levels: the species fed; the species did not feed), carcass covering overnight (two levels: covered; uncovered), dog presence at carcasses during daylight (two levels: present; absent) and avian scavenger guild type (two levels: ‘five-species guild’ and ‘three-species guild’) as the predictors. Third, we conducted a post hoc analysis to measure the tolerance of avian scavengers to domestic dog disturbance using our records on whether they fed on the carcasses that were visited by dogs during daylight (*n* = 12). Tolerance was treated as a dichotomous variable, assigning a ‘1’ when avian scavengers fed on a carcass that was visited by dogs during daylight and a ‘0’ when they did not. Avian scavenger records were classified into those that corresponded to the Andean Condor (i.e. the top scavenger), the vulture species (i.e. non-top obligate scavengers) and the caracara species (i.e. facultative scavengers). Subsequently, we fitted a GLMM with binomial distribution and logit link function, in which tolerance was included as the dependent variable, avian scavenger category (three levels; ‘condors’, ‘vultures’ and ‘caracaras’) as the predictor, and carcass as a random term. We estimated the effect size of the predictors of these models using Cohen’s *f*^*2*^ (i.e. the proportion of variance explained solely by the variable of interest relative to the total variance explained by all variables in the model) whose magnitude determined the effect size as non-significant (*f*^*2*^ < 0.02), small (0.02 ≤ *f*^*2*^< 0.15), medium (0.15 ≤ *f*^*2*^ < 0.35) or large (*f*^*2*^ ≥ 0.35) [42]. Computations were performed using R [43] in RStudio (version 2.3.4 [44]). A complementary summary of the fitted models can be found in electronic supplementary material, S2 (electronic supplementary material, table S3). Variables were deemed significant at *p* < 0.05.

## 3. Results

### 3.1. Carcass use

**Figure 2.**
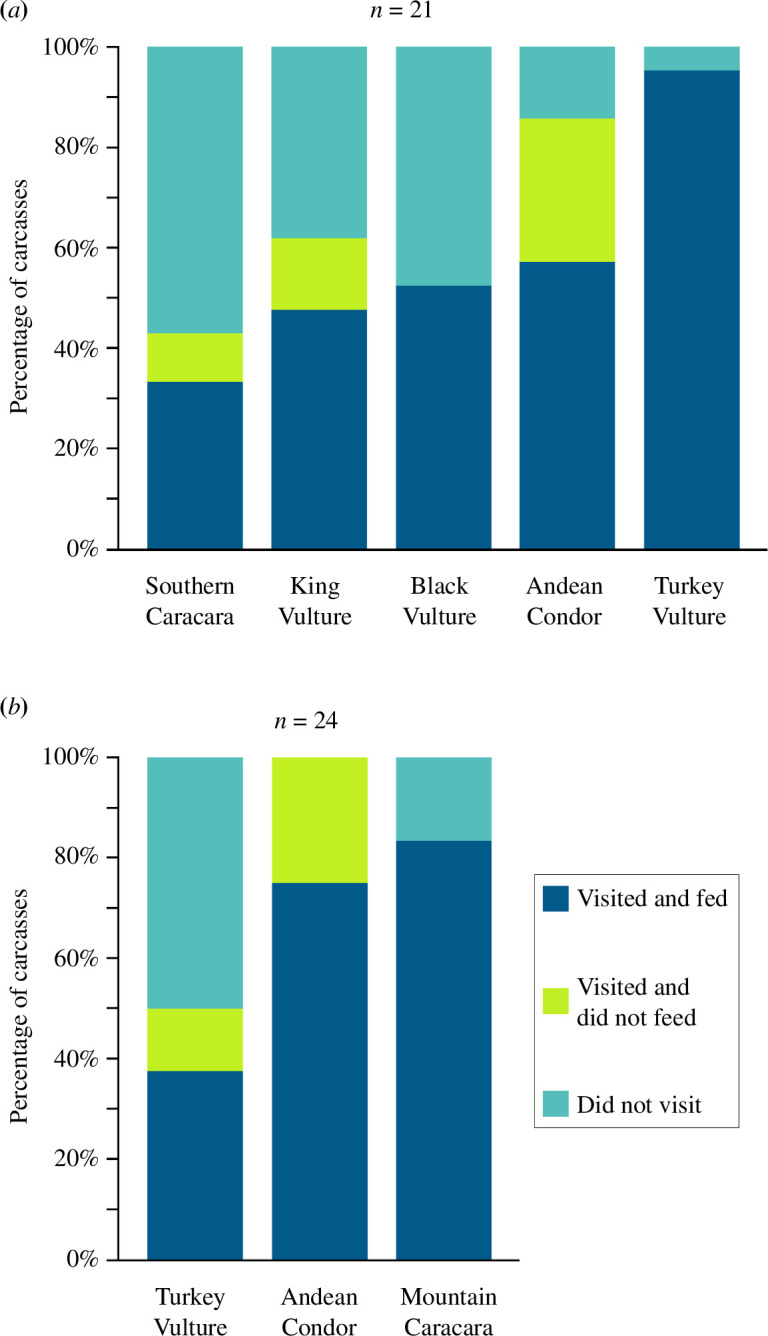
Frequency of use of the 45 carcasses by the studied avian scavenger species, in the five-species guild (*a*), and the three-species guild (*b*).

In the five-species guild and the three-species guild, avian scavengers used the carcasses in significantly different proportions (Fisher’s exact test, *p* < 0.001 in both guilds) (figure 2). Turkey vultures were the species that visited a higher number of carcasses in the five-species guild (20 carcasses, 95%), while Mountain caracaras did so in the three-species guild (20 carcasses, 83%); Andean condors were the second species that visited the most carcasses in both guilds: 12 carcasses (57%) and 18 carcasses (75%), respectively (see figure 2 for other species).

The proportions in which condors used the carcasses in the five-species guild and in the three-species guild were similar (Fisher’s exact test, *p* = 0.163), while in the case of Turkey vultures, it differed significantly (Fisher’s exact test, *p* < 0.001) (figure 2). See electronic supplementary material, S2.1 for complementary records on the frequency with which avian scavenger species were the first to detect and feed on carcasses.

### 3.2. Abundances

**Figure 3.**
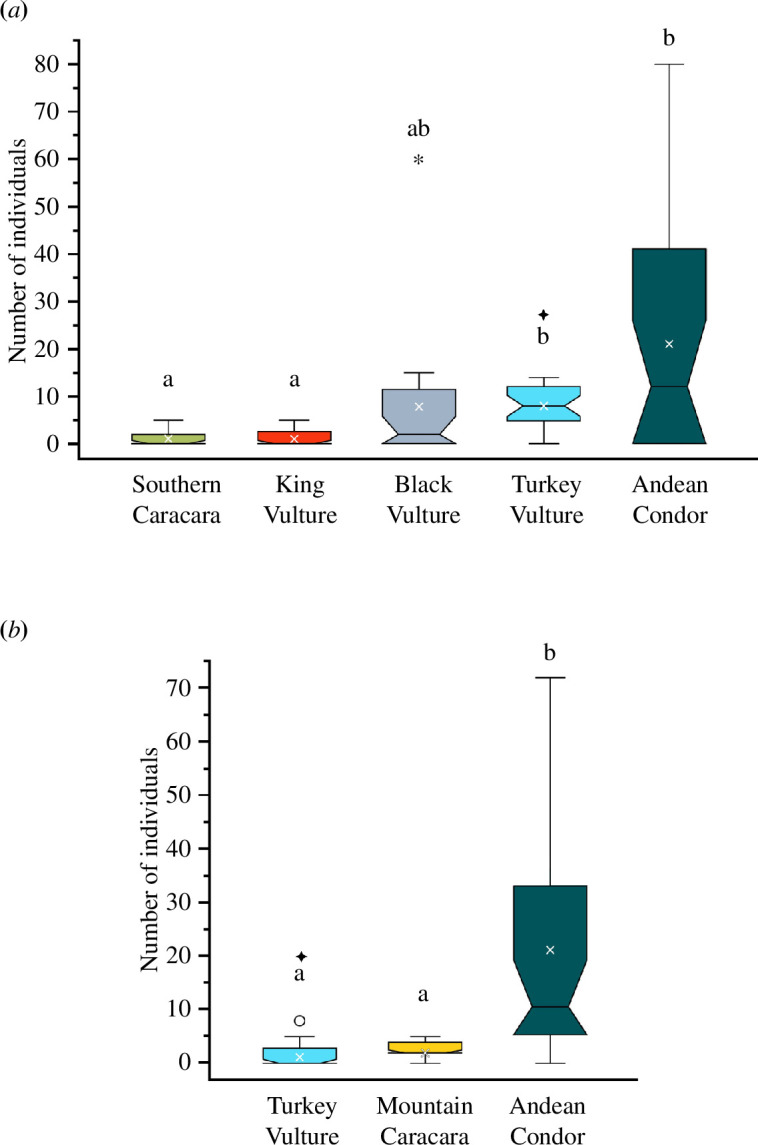
Average abundances (white crosses) of avian scavengers of the five-species guild (*a*) and the three-species guild (*b*). Non-matching letters indicate significant differences within each guild, while equal letters indicate no difference. A star mark indicates significant differences between guilds, and its absence indicates no differences. Outliers are represented with an asterisk (extreme), or an open circle (mild).

**Figure 4.**
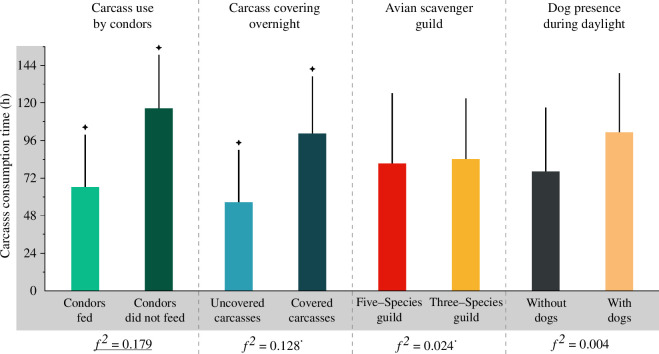
Average consumption time of the 45 placed carcasses depending on their use by Andean condors, their covering at night, their consumption by the studied avian scavenger guilds and their visit by domestic dogs during the day. Significant statistical differences are indicated by stars. Significant effect sizes of these variables are underlined (medium; 0.15 ≤ Cohen’s *f^2^* < 0.35), or marked with a superscript dot (small; 0.02 ≤ *f^2^* < 0.15).

In the five-species guild, condors were the most abundant species (x̅ = 21 ± 25 s.d. individuals per carcass), followed by Turkey (x̅ = 8 ± 4), Black (x̅ = 8 ± 13) and King (x̅ = 1 ± 2) vultures and Southern caracaras (x̅ = 1 ± 2) (Kruskal–Wallis: *χ*^2^ = 24.23, *df* = 4, *p* < 0.001) (figure 3). In the three-species guild, condors were also the most abundant species (x̅ = 21 ± 21 s.d. individuals per carcass), followed by Mountain caracaras (x̅ = 2 ± 1) and Turkey vultures (x̅ = 1 ± 2) (Kruskal–Wallis: *χ*^2^ = 17.5, *df* = 2, *p* < 0.001) (figure 3). Condors’ abundance in the five-species guild was similar to that in the three-species guild (Mann–Whitney test, *U* = 231, *p* = 0.637), while Turkey vultures’ abundance in the five-species guild was significantly higher than in the three-species guild (Mann–Whitney test, *U* = 47.5, *p* < 0.001) (figure 4).

### 3.3. Carcass consumption time

For results and discussion on carcass detection time and carcass consumption start time, see electronic supplementary material, S2.2. Across carcasses (*n* = 45), carcass consumption time was significantly shorter when condors fed (x̅ = 66.22 h ± 33.55 s.d.; 30 carcasses) than when they did not (x̅ = 116.32 ± 34.34; 15 carcasses) (GLM: *β* ± s.e. = 0.007 ± 0.002, *t* = 4.094, *p* < 0.05) (figure 4). Likewise, it was shorter when carcasses were not covered overnight (x̅ = 56.61 h ± 33.35 s.d.) than when they were (x̅ = 100.46 ± 36.47) (GLM: *β* ± s.e. = 0.008 ± 0.002, *t* = 3.612, *p* < 0.001); whereas type of avian scavenger guild (GLM: *β* ± s.e. = 0.000 ± 0.002, *t* = −0.228, *p* = 0.821) and dogs’ presence during daylight (which was recorded at 12 carcasses, 26.67%) (GLM: *β* ± s.e. = −0.003 ± 0.002, *t* = −1.767, *p* = 0.084) were not influential factors (figure 4).

**Figure 5.**
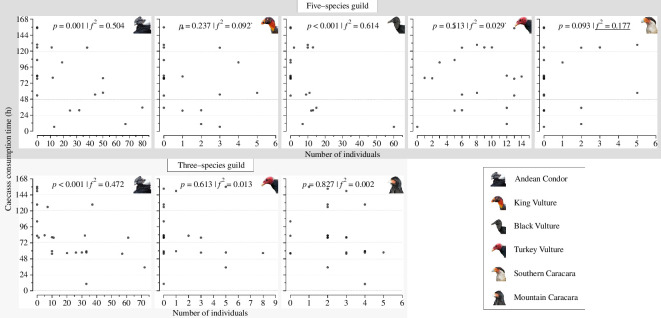
The relationship between the abundance of the studied avian scavengers and the consumption time of the placed carcasses. Statistically significant relationships are in bold. Significant effect sizes of the abundances are in bold (large effect sizes; Cohen’s *f^2^* ≥ 0.35), underlined (medium; 0.15 ≤ *f^2^* < 0.35) or marked with a superscript dot (small; 0.02 ≤ *f^2^* < 0.15).

Overall, the species whose abundances were most influential on carcass consumption time were the Andean Condor and the Black Vulture in the five-species guild, and the Andean Condor in the three-species guild (figure 5). In the five-species guild, carcass consumption time tended to be shorter the greater the abundances of condors (GLM: *β* ± s.e. = −0.966 ± 0.247, *t* = −3.903, *p* < 0.05) and Black vultures (GLM: *β* ± s.e. = − 1.385 ± 0.283, *t* = −4.886, *p* < 0.05). On the other hand, carcass consumption time tended to be longer the greater the abundance of Southern caracaras (GLM: *β* ± s.e. = 10.460 ± 5.830, *t* = 1.794, *p* = 0.093), whereas its relationship with the abundance of Turkey (GLM: *β* ± s.e. = −1.377 ± 2.058, *t* = −0.669, *p* = 0.513) and King (GLM: *β* ± s.e. = −5.724 ± 4.651, *t* = − 1.231, *p* = 0.237) vultures was not straightforward (figure 5). In the three-species guild, carcass consumption time tended to be shorter the greater the abundance of condors (GLM: *β* ± s.e. = −1.150 ± 0.272, *t* = −4.231, *p* < 0.05), while its relationship with the abundances of Mountain caracaras (GLM: *β* ± s.e. = −1.247 ± 5.622, *t* = −0.222, *p* = 0.827) and Turkey vultures (GLM: *β* ± s.e. = 1.691 ± 3.295, *t* = 0.513, *p* = 0.613) was unclear (figure 5).

### 3.4. Avian scavenger tolerance to dog disturbance

Tolerance to dog disturbance depended on the avian scavenger category (Cohen’s *f^2^* = 0.475), inasmuch as condors visited 10 of the 12 carcasses visited by dogs during daylight and fed on 4 (40%) of them, significantly less than the other two avian scavenger categories (non-top obligate scavengers and facultative scavengers) (GLMM: *χ^2^* = 6.79, *df* = 2, *p* < 0.05). Indeed, the other vulture species together visited 10 carcasses that were also visited by dogs during daylight, and fed on 9 (90%) of them and the two caracara species together visited 6 carcasses visited by dogs during daylight, and fed on 5 (83%) of them.

## 4. Discussion

We found that the Andean Condor is a pivotal scavenger species in the consumption of large carcasses, both by virtue of its presence and higher relative abundance when feeding, playing a leading role in reducing carcass consumption time. It significantly influenced carcass consumption in both the five-species and the three-species guild while being the most cautious species before feeding, and the most sensitive towards domestic dogs, but also being the one that fed most frequently (figure 2). These findings support the hypothesis that the Andean Condor is a top scavenger in the ecosystems in which it is present.

The consumption time of the carcasses that condors fed on was, on average, 1.75 times shorter than that of those on which they did not (figure 4). Moreover, the greater abundance of feeding condors shortened carcass consumption time more than a greater abundance of any other avian scavenger species by 1.65 to 5.96 times on average (figure 5). Therefore, our study demonstrates the key functional role of condors in accelerating the ecological process and ecosystem service of carrion removal. This was true despite their well-known wariness in deciding to land and feed [7,26], meaning they took up to almost three days to do so (electronic supplementary material, figure S2,3). In this regard, we quantified that the probability that condors decide to feed on a single large carcass was between 1.15 and 1.5 times lower than that of co-occurring avian scavengers, even when condors were the first species to detect the carcass (electronic supplementary material, figure S2,3). Additionally, we measured such behaviour in terms of how long it took condors to begin feeding after detecting a carcass and found that, on average, they were 1.58 to 3.8 times slower than any other avian scavenger species, except for Black vultures, which took virtually the same time (electronic supplementary material, figure S3).

Among Neotropical vultures, Andean condors and Black vultures are the only ones that exhibit a marked tendency towards conspecific grouping to feed, thereby, similar foraging strategies between both species can be expected [8,45]. Importantly, we found that both had a predominant effect on carcass consumption since, with a greater abundance of feeding condors and Black vultures, the consumption time of the carcasses was significantly shorter (figure 5; electronic supplementary material, figure S4). This can be explained because, unlike the other avian scavenger species we studied, these two can form large foraging groups that can quickly consume a carcass in a feeding frenzy [7,8]. For the Andean Condor, this feeding strategy appears as the usual one when feeding on large carcasses [37], while for the Black vulture, we only observed it once (figure 5; electronic supplementary material, figure S4). Based on these observations, the Black vulture could be categorized as a top scavenger on par with the Andean Condor, yet relevant functional characteristics differ between the two species (table 1).

First, Black vultures are not capable of completely opening a large carcass (e.g. bovine, equine); therefore, without the prior intervention of condors or other animals that can do so, they would not be able to consume a carcass of this type, even if they were in large foraging groups [7,8]. Second, Black vultures can outcompete condors at carrion sources only if they are found in large enough numbers [46]; thus, following what we observed in the field, the ecological relationship between both species can be defined as a continuum between commensalism or trophic facilitation (i.e. a few condors open and feed on a large carcass allowing Black vultures to access it) and competition (i.e. large numbers of either condors or Black vultures displace the others from a large carcass). Third, Andean condors have some of the largest home ranges known in terrestrial vertebrates [47], which can be at least six times larger than the known home ranges of Black vultures [48]; therefore, its influence on scavenging can be exerted across vast areas and ecoregions, larger than what any other scavenger species could probably cover [47,49]. In sum, although both species are able to rapidly consume a carcass and, in those terms, have the potential to be top scavengers, only the Andean Condor can be such thanks solely to the advantage conferred by its phenotype (table 1).

Strikingly, we found no significant differences in the consumption time of carcasses visited by dogs and those that were not visited (figure 4). We believe this was because the dogs’ visits to the carcasses (always a single visit per carcass) occurred in the last days of consumption. Thus, we assume that their effect was limited since in all cases there was only one disturbance event for avian scavengers when they had already started to consume the carrion or when less of it remained. Nonetheless, we found base-line evidence of a gradient of tolerance to dogs by Andean avian scavengers (ordered from highest to lowest tolerance: vultures > caracaras > condors), in which all species, except the condor, seem to cope with this disturbance so that their role as scavengers is not affected. Such a gradient corroborates what has been recorded elsewhere in the Americas regarding extinction risk in avian scavengers, whereby the largest species (i.e. condors) generally appears as the least tolerant not only to dogs but to disturbances in general, and, therefore, the most vulnerable [13,30,46,50]. Put another way, top scavengers like the Andean Condor, which we establish here as a model, constitute conservation priorities both because of the prominent ecological role they play and the intrinsic vulnerability they may have [8,30,51,52]. A complementary discussion of the studied factors affecting carcass consumption time is provided in electronic supplementary material, S2.3.

With scavengers—most importantly vultures—and scavenging in jeopardy worldwide [53,54], competition for food with domestic dogs, plus injuries or death from dog attacks, must continue to be evaluated. Following this line and focusing on optimizing management efforts, special attention should be paid to the fact that the persistence of unattended domestic dogs strongly depends on accessible, predictable and abundant food sources [55], which in turn can be used as avian scavenger monitoring and conservation tools (i.e. vulture feeding stations) [56]. Vultures/condors may be well adapted to tolerate feeding stresses (e.g. having less and less access to carcasses because of dog activity) by being able to consume large amounts of food at once and to go for comparatively long periods of time without feeding [7,8]. However, food scarcity and the availability of easily accessible low-quality food (e.g. waste dumps) can still represent a problem for the subsistence of their populations [6,9,57]. In this regard, interconnected research paths dealing with ecological and conservation questions should be followed [30], prioritizing the study of overlooked topics that—like the search for appropriate identification of top scavengers—can have rapid and significant repercussions on how we understand and conserve a fundamental component of the biosphere, as are scavenger assemblages and the ecosystem service they deliver.

We suggest that top scavengers could be common currency to compare ecosystems with their own scavenger communities and contrast their functioning across different environmental gradients or geographic regions, facilitating the study of how a similar ecological function is achieved by different organisms in different conditions, and helping to clarify our interpretation of the scavenging process [53,54]. Here we demonstrated how a top scavenger—the Andean Condor—actually drives the scavenging process, being a true linchpin within its guilds. Elaborating on the top scavengers’ approach can be a valuable tool, as it offers a simplified but meaningful way to address many questions in carrion ecology (e.g. what role do top scavengers play in linking small-scale scavenging processes to large-scale scavenging patterns? What role do top scavengers play in structuring scavenger assemblages? [58], what approaches should be used to quantify top scavengers’ specific contributions to ecosystem functioning and to predict possible changes if they disappear? [59]). In using this tool, the extensive and detailed work that has been conducted on top predators could serve as a model [1]. It will be important to remember that the matter of top scavengers should be factual and that scavengers’ ecological niches should continue to be methodically studied rather than assumed.

## Data Availability

All data used in this work are attached as supplementary material [60].
